# Intracellular Demography and the Dynamics of Salmonella enterica Infections

**DOI:** 10.1371/journal.pbio.0040349

**Published:** 2006-10-17

**Authors:** Sam P Brown, Stephen J Cornell, Mark Sheppard, Andrew J Grant, Duncan J Maskell, Bryan T Grenfell, Pietro Mastroeni

**Affiliations:** 1 Department of Zoology, University of Cambridge, Cambridge, United Kingdom; 2 Section of Integrative Biology, University of Texas at Austin, Austin, Texas, United States of America; 3 Institute of Integrative and Comparative Biology, University of Leeds, Leeds, United Kingdom; 4 Department of Veterinary Medicine, University of Cambridge, Cambridge, United Kingdom; 5 Center for Infectious Disease Dynamics, Pennsylvania State University, University Park, Pennsylvania, United States of America; 6 Fogarty International Center, National Institutes of Health, Bethesda, Maryland, United States of America; Princeton University, United States of America

## Abstract

An understanding of within-host dynamics of pathogen interactions with eukaryotic cells can shape the development of effective preventive measures and drug regimes. Such investigations have been hampered by the difficulty of identifying and observing directly, within live tissues, the multiple key variables that underlay infection processes. Fluorescence microscopy data on intracellular distributions of Salmonella enterica serovar Typhimurium (*S.* Typhimurium) show that, while the number of infected cells increases with time, the distribution of bacteria between cells is stationary (though highly skewed). Here, we report a simple model framework for the intensity of intracellular infection that links the quasi-stationary distribution of bacteria to bacterial and cellular demography. This enables us to reject the hypothesis that the skewed distribution is generated by intrinsic cellular heterogeneities, and to derive specific predictions on the within-cell dynamics of *Salmonella* division and host-cell lysis. For within-cell pathogens in general, we show that within-cell dynamics have implications across pathogen dynamics, evolution, and control, and we develop novel generic guidelines for the design of antibacterial combination therapies and the management of antibiotic resistance.

## Abstract 2

Modeling of infection shows that heterogeneities in bacterial burden per host cell need not be explained by differences in host cell permissiveness; this has important implications for understanding the ecology of infection.

## Introduction

Understanding the within-host proliferation dynamics of microbial pathogens is a challenge of clear medical importance, underlying many details of pathogenicity, transmission, and pathogen evolution [1]. However, current modelling frameworks for bacterial infections offer little or no resolution on the localisation of bacteria within the host. Some generic microparasitic models track the within-host intensity of infection [2–4], but internal dynamics in these models remain averaged at the whole-body level. New technologies are now giving insights into bacterial dynamics on the intracellular level, moving beyond the current predictive abilities of existing theoretical models. We present what is, to our knowledge, the first within-host model to trace explicitly bacterial proliferation dynamics on both the within- and among-cell levels. The increased resolution of our within-host demographic model presents a powerful framework linking individual microbe behaviour (division, host lysis, extracellular survival) with in vivo infection dynamics (bacterial population growth rate and distribution). This general framework enables the generation of numerous testable hypotheses spanning mechanistic interventions (e.g. drug treatments, vaccines) and their dynamical effects (e.g. bacterial persistence or clearance).

Salmonella enterica are Gram-negative bacteria that infect a range of animals, resulting in a broad spectrum of disease. S. enterica serovar Typhi (*S*. Typhi) causes 22 million cases of typhoid fever in humans per year, resulting in at least 200,000 deaths [5]. Other serovars (e.g. *S.* Typhimurium*, S.* Enteritidis) infect domestic animals and humans presenting a serious concern for the food industry [6]. *S.* Typhimurium infections in mice (mouse typhoid) have been studied extensively, allowing a range of infections with varying degrees of severity. Mouse typhoid models form the basis of the understanding of pathogenesis and immunity in systemic salmonellosis and a reference for understanding the biology of several other bacterial infections. The pathogenesis of salmonellosis is strictly related to the dynamic interactions between bacteria and phagocytic cells at different body sites. Intracellular bacterial growth within phagocytes is restrained via diverse mechanisms such as those requiring reactive oxygen intermediates (ROI) and reactive nitrogen intermediates (RNI), lysosomal enzymes, and defensins [7–9]. At the same time, S. enterica has evolved sophisticated mechanisms to prevent the targeting of antibacterial compounds to the S. enterica-containing vacuole (SCV) [10–11]. The ability to grow inside infected phagocytes and to avoid killing is only one of the prerequisites for S. enterica virulence. Escape from infected macrophages and dissemination to other uninfected cells is a further necessary step in S. enterica proliferation. While much is known about mechanisms of induction of cell death by S. enterica serovars in cells in culture [12], very little is known about the parameters and mechanisms (e.g. necrosis vs. apoptosis) that govern the escape of S. enterica from infected cells, in vivo in the host animal, and how they contribute to the spread of the bacteria to uninfected cells in host organs. Very little is known on whether and how permissivity to bacterial replication of individual host cells and bacterial and host genetics can affect the parameters of spread and distribution of S. enterica in the host. We have recently used multicolour fluorescence microscopy (MCFM) and laser scanning confocal microscopy (LSCFM) to visualise individual bacteria in vivo, localised within host phagocytes. We showed [7] that the growth of S. enterica in the tissues of infected animals results in the continuous spread of the micro-organisms to new host cells and foci of infection rather than simply increased bacterial numbers within the initial ones. This results in typically low numbers of bacteria in each infected phagocyte and in increases in the number of infected phagocytes that largely parallel the net rate of bacterial growth of the micro-organisms in the tissues.

**Figure 1:**
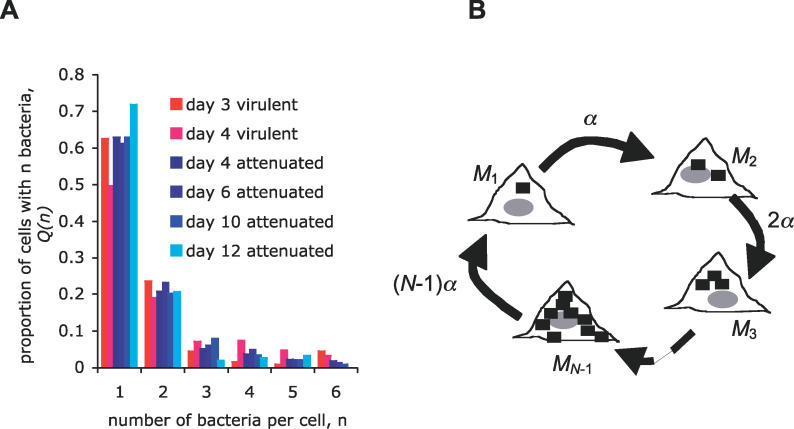
Within-Host Distribution and Growth of Salmonellae (A) Distributions of bacterial charges, *Q_n_,* during the course of infection. Data [7] for “virulent” (SL5560, red shades) and “attenuated” (SL3261, blue shades) infections. Bacteria contained within CD18^+^ phagocytic cells were visualized by immunostaining tissue sections from infected mice. (B) Threshold burst model schema. Triangles represent a host cell through four moments in time. First, the host cell is infected with a single bacterium (rectangle). This bacterium divides at rate *α*, entailing a transition from single *(M*_1_ *)* to double *(M*_2_ *)* infection. The transition from *M*_2_ to *M*_3_ occurs at rate 2*α* (assume equal and constant division rate for each bacterium, i.e. asynchronous division). Subsequent transitions from *M_n_* to *M_n_*_+1_ occur at rate *nα,* up to final division (at rate (*N*−1)*α*) when the host cell bursts to release *N* bacteria which instantly infect *N* new host cells.

In Figure 1A, the variation in intracellular bacterial counts within CD18^+^ phagocytes in mouse liver sections is illustrated for both virulent (SL5560) and attenuated (SL3261) strains, during the course of infection. Similar results were obtained from observations of infected spleens (unpublished data). The consistency of the bacterial distribution within phagocytes in all experiments is striking. While the distributions of intracellular bacterial numbers are similar over time, the virulent and attenuated strains differ greatly – and predictably – in their net overall growth rates [7]. Thus while the population growth rate *γ* is a consistent 0.09 *h* ^−1^ for the virulent strain, that of the attenuated strain peaks at only *γ* = 0.03 *h* ^−1^.

The observation of a consistent skew in bacterial count per host cell, irrespective of net bacterial growth rate and time since infection (Figure 1A), has led to the inference that the observed heterogeneity in intracellular bacterial numbers reflects a heterogeneity in host cell permissiveness [6–7]; more specifically that lightly infected cells are resistant and more heavily infected cells are permissive to bacterial replication. This inference is plausible given the known vertical (differentiation stage) and horizontal (anatomical source) heterogeneity within phagocyte populations [13]. Our model framework shows that this inference is unnecessary, since a simple demographic model within a homogenous population of host cells results in a constantly skewed distribution of bacterial counts. However, the fact that it is unnecessary to invoke heterogeneity to explain, in general, skewed distributions of bacteria within host cells does not rule out a role for host cell heterogeneity in shaping the precise quantitative distributions observed in our infection systems. We therefore explore, within this novel framework, the potential role of intrinsic versus stochastic variability amongst host cell populations in shaping the specific observed distributions of bacteria among cells. We use existing distributional data obtained from the direct observation of bacterial populations in the tissues of mice systemically infected with S. enterica to reject the intrinsic heterogeneity hypothesis, and then use the favoured stochastic model to derive predictions on the within-cell dynamics of *Salmonella*. Finally, we use our analysis of within-cell and among-cell behaviour of *Salmonella* to derive generic predictions on the relative efficacy of using therapeutic regimes that comprise different combinations of antibiotics that can or cannot gain access to the host intracellular compartment.

## Results

### Bacterial Distributions in a Homogeneous Host Cell Population

To capture the dynamics of bacterial proliferation on both the within- and among-host cell levels, we use a “branching process” model [14–15] governed by bacterial division rate *α* and host cell burst-size *N* (Figure 1B, Methods). A branching process describes the stochastic evolution of the numbers of descendents arising from a given number of ancestors. We focus on simple branching processes where the initial ancestors and all descendents reproduce independently with the same probability function. To incorporate among-cell spread, we define a burst size, *N*, where the host cell lyses liberating *N* bacteria. Based on the vast excess of non-infected cells in the host tissues at the observed times of infection we assume that each released bacterium instantaneously infects a separate new host cell. Thus, the accumulation of bacteria within a host-cell can occur only by binary fission, governed by division rate *α.* The assumption of a rapid infection of phagocytes due to an excess of host cells is consistent with the very rapid blood clearance of circulating bacteria and the rapid uptake of *Salmonella* by cultured phagocytes [10,16,17].

When *N* is fixed, we have an extremely simple model governed by two parameters, division rate *α* and burst size *N* (Figure 1B). Given the assumption of instantaneous infection following host cell burst, the bacterial population growth rate *γ* is determined solely by the division parameter α with *γ = α*. In this model we find that the proportion of host cells *Q_n_* with *n* bacteria rapidly approaches a distribution that is independent of division rate and time – around 50% of infected cells contain only one bacterium, whereas some cells are heavily infected (Equation 2, Methods). It is therefore not necessary to invoke intrinsic heterogeneity in host cell permissiveness to explain heterogeneity in bacterial count: heavily infected cells are not necessarily any more or less permissive than lightly infected cells, since the heterogeneity can be simply a result of different times since host cell infection.

**Figure 2:**
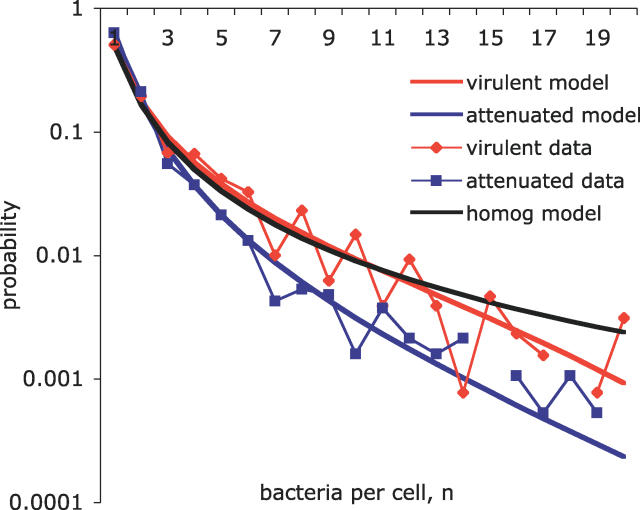
Observed and Predicted Distribution of Bacterial Charges, *Q_n_*, with Data Pooled across Time-Points Red dots represent virulent data, blue dots represent attenuated data. The black line corresponds to the fixed burst model (Methods, Equation 2 ) with large burst size *N*. The red line is the best fit to the virulent data and the blue line is the best fit to the attenuated data (density-dependent fission model, parameter values in Table 2).

Given the consistency of bacterial distribution throughout the exponential phase of bacterial growth (Figure 1A), we combine the per-day distributional data into a single dataset for each strain of bacteria, and contrast these combined (time-independent) distributions with the predicted distributions of the fixed-burst model, under the assumption of a large burst size. Both datasets show a clear qualitative agreement with the model (black curve, Figure 2), capturing the modal charge of one, the monotonic decrease in frequency with increasing charge, and the distributional invariance with time. The homogenous model illustrates that heterogeneities in bacterial numbers per host cell can follow from an entirely homogeneous population of host cells. However, it is still possible that significant heterogeneities among host cells exist, and have an influence on the quantitative distribution of intracellular bacterial numbers per cell. In the following two sections, we explore competing hypotheses that introduce variability into the cell burst size, either by intrinsic cell heterogeneity or by noise in the stochastic burst process.

### Constitutive Heterogeneity

In the homogeneous model in the previous section, we assumed a single lysis threshold *N*, and find a qualitative agreement for the single threshold model with both the virulent and attenuated data (Figure 2). What if, in contrast to the single threshold model, cells differ in their intrinsic response to infection? Given that S. enterica resides in both macrophages and polymorphonuclear phagocytes during the exponential growth phase of the infection [6], this is a reasonable possibility. Introducing an intrinsic host-defined bimodality to intracellular division rate does not improve the model fit (unpublished data), however, introducing a bimodality to the burst threshold of host cells leads to a significant increase in model performance over the single threshold model (Table 1). The bimodal threshold model also out-performs a Poisson distributed threshold model, but is in turn out-performed by a series of two-parameter distributions (normal, gamma, negative binomial), introducing an additional “shape” parameter (Table 1, Methods, Protocol S1). The two-parameter models gave similar fits, all supporting the conclusion of a significantly inflated variance in burst threshold.

**Figure 3:**
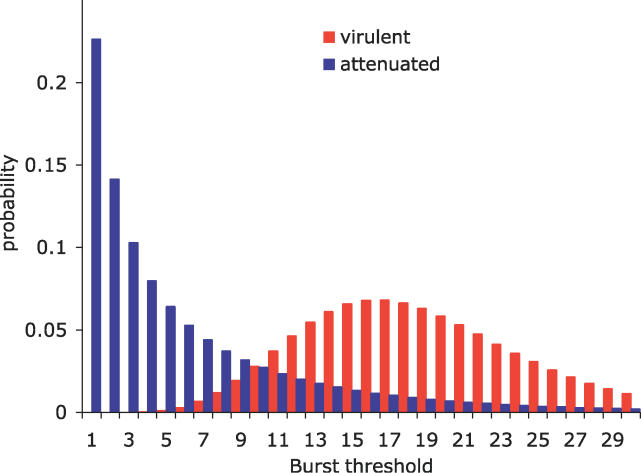
Burst Threshold Distributions Inferred from stationary distribution of bacterial charges, assuming a gamma distribution of burst thresholds (parameter values in Table 1).

The gamma model offers the best phenomenological fit for both the virulent and attenuated datasets (Table 1), allowing us to infer the distribution of burst thresholds (Figure 3) from the observed stationary distribution of bacterial counts (Figure 2). In Figure 3, we can see that the distinct parameterisations predict a clear difference in the distribution of burst thresholds between cells containing the virulent or attenuated bacterial strains, with cells containing the virulent strain typically bursting at higher bacterial densities. In the next section, we explore mechanistic hypothesis accounting for these strain differences, linking host cell lysis to the number of bacteria within a host-cell, and their rate of division.

### Stochastic Variability

So far, we have assumed that cells are programmed to burst the instant that the within-cell bacterial quorum reaches a predetermined threshold *N*, where *N* may vary between cells. In this section, we develop an alternate hypothesis: that cells are intrinsically identical, but lysis is itself a stochastic process which takes place at a rate *μ*. Cell burst size will then be variable as a result of stochastic noise in the lysis process. We use the observed distributions of bacterial counts (Figure 2) to infer putative underlying processes of microbial division and lysis. As before, we assume that after lysis all bacteria instantaneously reinfect, so the epidemic growth rate within the host again equals the bacterial division rate, *γ=α* (provided *α* is constant).

**Table 1:**
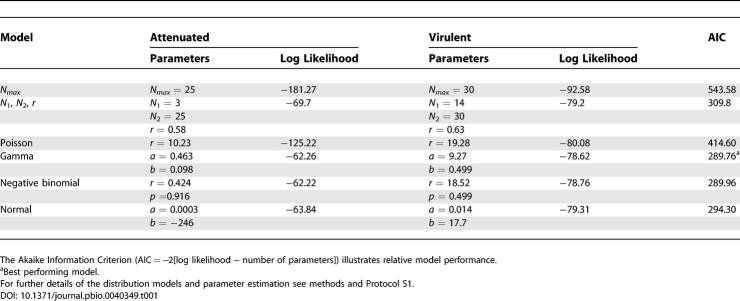
Maximum Likelihood Parameter Estimates and Model Selection, Threshold Burst Models

Given any non-zero lysis rate *μ* we find that reducing *α* not only reduces the population growth rate *γ* but also biases the distribution towards lower intensities of infection, as seen in the attenuated strain (Figure 2). This distributional bias can be understood as a consequence of an increase in the relative exposure to host cell lysis; slower-growing bacteria take longer to reach a given size, so the probability of lysis before reaching that size will be greater.

To explore a range of potential intracellular behaviours (e.g. division rate and host cell lysis depend on bacterial density within a cell), we introduce density-dependence to the burst rate *μ* and division rate *α* parameters. We now assume that host cells are lysed and release their bacteria in a variable and potentially density-dependent manner, with the rate of lysis given by *μ_n_* = *μ*_0_ *+μ*_1_ *n + μ*_2_ *n^2^.* Furthermore, we assume that bacteria divide at a rate dependent on the density of bacteria within a cell, with the rate of division given by *α_n_* = *α_0_ e^−α_e_n^*. The *α_e_* term thus represents the strength of density-dependent reduction in division rate with increasing within-cell density of bacteria. We again assume that after lysis all bacteria instantaneously reinfect, so now the population growth rate *γ* reflects a weighted summary of intracellular division rates *α_n_* (while no explicit solution of *γ* can be found, it can be computed numerically from the other parameters).

We find that the best-supported model combines density-independent lysis (*μ*_0_) with density-dependent division (*α_e_* > 0) (Table 2). Again, we find notable differences between the virulent and attenuated strains, with the virulent strain characterised by a greater density-dependence in its intracellular division rate. This model has an AIC score which is lower than that of the best constitutive heterogeneity model by 2.22, which implies an improvement in model support by a factor of e^2.22^ > 9.2. The best-fitting model makes important predictions about the intracellular behaviour of S. enterica. This model predicts a density-dependent slowing in intracellular bacterial division rate and a constant rate of stochastic bursts in infected phagocytes, independent of intracellular bacterial numbers.

**Table 2:**
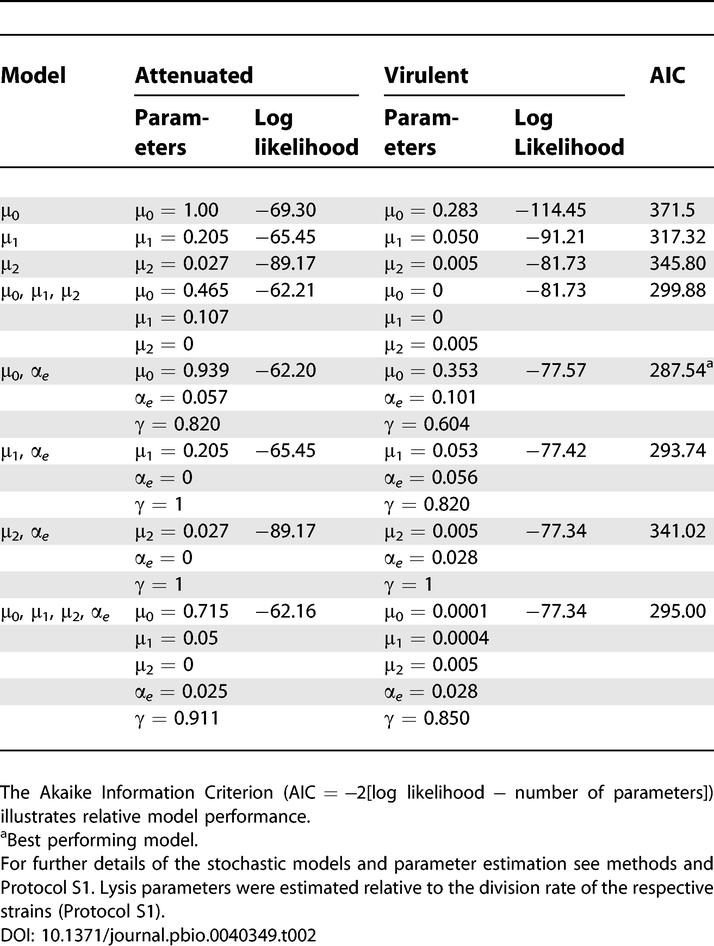
Maximum Likelihood Parameter Estimates and Model Selection, Stochastic Burst Models

### Medical Intervention

We can use this novel picture of within- and among-cell infection dynamics to shed new light on the within-host dynamics of bacterial control; first, though, we need to add an extra biological refinement. In the above models, we assume that the bacteria released upon the burst of an infected cell are viable, and that they all create new infections. In the early stages of primary infections the relative insignificance of extracellular mortality is borne out by three independent lines of evidence: first, published estimates of mean division-rate *α* and population growth rate *γ* in murine salmonellosis are in close agreement [18]; second, *Salmonella* are resistant to the bactericidal effect of serum [6]; third, fitting an extracellular survival parameter to the best-fitting model above (density-dependent fission) results in no change to the maximum likelihood (unpublished data). Even in the presence of an antibody response (later stages of primary infections and secondary infections) extracellular mortality is likely to be negligible. In fact, S. enterica is not killed by exposure of antibody and complement in the extracellular compartment and antibody does not affect the course of primary systemic salmonellosis [19]. However, extracellular survival is potentially of great importance when we turn to considering the medical control of infection as antimicrobials can kill extracellular bacteria.

Here we introduce an extracellular survival term *β*, so that a cell that bursts with *N* bacteria leads to *βN* new infections; the prior models result when *β* = 1. We can now consider the relative merits of “*α*_0_ interventions” (to reduce average intracellular proliferation, *α_n_* = *α*_0_ *e^−αen^*) and “*β* interventions” (to reduce extracellular survival *β*). *α*_0_ and β are merely axes describing the potential space of antibiotic action – mechanistic constraints ensure that not all combinations of *α*_0_ and *β* efficacy will be possible, in particular an antibiotic that is only active intracellularly is not currently available. Nonetheless this treatment duality covers an important, unexplored dimension of existing and potential antibiotic treatments of S. enterica (or other microbial diseases) – from the exclusively extracellular killing of antibiotics such as gentamicin to the combined intracellular and extracellular killing of antibiotics such as ciprofloxacin that efficiently penetrate, and indeed are concentrated into eukaryotic phagocytic cells [20]. What are the dynamical consequences of these interventions?

The simplest yardstick is to look at the impact of a given intervention on the within-host reproductive number, *R*_0_. *R*_0_ is the mean number of newly infected cells, following introduction of a single infected cell into a population of susceptible cells in the absence of medical interventions [21]. Thus in our framework, *R*_0_ is equivalent to the mean burst size 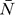 , and the effective reproductive number *R* (given medical intervention) is equivalent to *β* 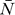 (*α*_0_)*.* When *R* < 1, the infection cannot spread, thus we have control when *β* < 1/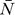 (*α*_0_)_._

**Figure 4:**
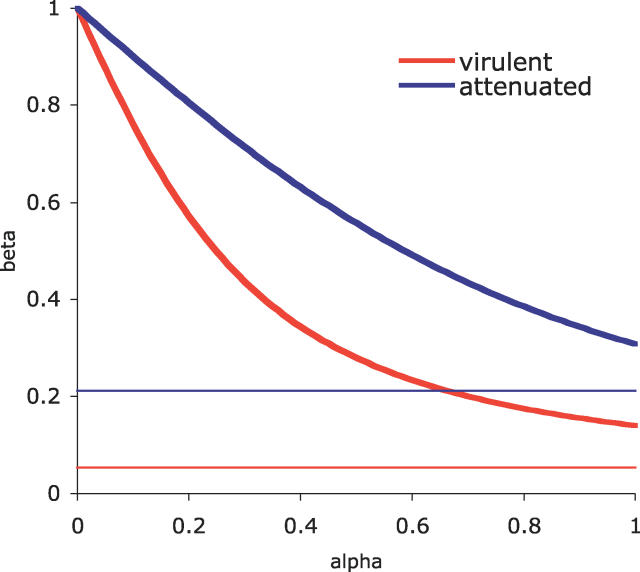
Extracellular versus Intracellular Control of Within-Host Proliferation Within-host control contours (reproductive number *R* = 1) as a function of intracellular division parameter *α*_0_ (normalised to a maximum of 1) and extracellular survival probability *β.* Thick lines are control contours assuming the density-dependent fission model (parameter values in Table 2). Thin lines are control contours assuming the gamma-distributed threshold model (parameter values in Table 1).

In Figure 4, control contours (*R* = 1, see Methods) are plotted as a function of *α*_0_ and *β* for both the virulent and attenuated strain parameterizations, below these contours, infections are fated to go extinct. The curved lines correspond to the control contours for the best fitting density-dependent division models (Figure 2, Table 2). In the absence of any intracellular control (*α*_0_ = 1), we find that the infection can be controlled by reducing *β* below 1/ *R*_0_ (intersections of blue and red curves with *α*_0_ = 1 in Figure 4), and that extracellular *β* control is more feasible for the attenuated strain, due to its lower *R*_0._ In contrast, in the absence of extracellular control (*β* = 1), control can only be achieved if intracellular growth becomes negative (i.e. intracellular killing outweighs intracellular proliferation), irrespective of the virulence of the strain.

Turning to combined interventions, for the stochastic lysis models we see that the *β* control target can be reduced by increasing *α*_0_ control, as reducing the rate of intracellular division will lead to a reduced mean burst size 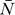 , and therefore a greater sensitivity to extracellular control (Figure 4). Thus our analysis predicts that the efficacy of common extracellular antibiotics can be enhanced by supplementation with antibiotics slowing intracellular bacterial division. This implies that both bacteriostatic and bactericidal drugs can potentiate the therapeutic efficacy of extracellular antibiotics. The predicted density-dependent slowing in within-cell division suggests that interventions causing a delay in host cell lysis would be particularly beneficial in slowing the spread of an infection, by decreasing the mean rate of intracellular proliferation.

It is worth noting that the prediction that decreasing *α*_0_ will favour *β* control is sensitive to the assumption of the stochastic model that cells lyse at a rate *μ_n_,* implying a constant mean time to lysis (defined by the parameterisation of *μ_n_*), and therefore a smaller burst given a lower rate of intracellular division. In contrast, under the threshold model, we assume that cells lyse at a predetermined density *N*, thus decreasing the intracellular growth rate (in the range 1 > *α*_0_ > 0) will not affect *N* (only affecting the time to *N*) and therefore will have no consequence for the threshold to extracellular control (gamma distributed threshold model, thin lines, Figure 4). Examining the dynamical response to various combined antibiotic therapies would provide further discriminating tests of the various demographic models developed above.

## Discussion

Our first finding is that it is not necessary to invoke heterogeneities in host-cell response in order to explain the consistent heterogeneities in bacterial numbers per host cell. An extremely simple model of bacterial proliferation through a homogeneous population of host cells can account for the key qualitative features of in vivo bacterial spread, and even offer an impressive quantitative fit to the data on the distribution of virulent, but not attenuated bacteria. (black line, Figure 2).

The key underlying assumption is that there is a sustained excess of host cells that can be infected, which can account for the secondary assumptions of an instantaneous absorption of newly-released bacteria and no multiple infection (i.e. accumulation of bacteria only by within-phagocyte division). The assumption of a large excess of host cells ensures a constant epidemic spread of the infectious agent.

Testable predictions can be generated by examining violations of this assumption. If (as in the case of a late acute infection) host cells become scarce, the supply of new (singly infected) host cells will diminish, and the distribution of bacterial counts will become more skewed towards higher counts. Similar non-equilibrium predictions can be made for the very early stages of the infectious cycle, where initially infected host-cells have not yet burst. During this early phase, the mean within-host intensity begins at one, then peaks above the static distribution before bursts begin to redistribute bacteria among new host cells. Predictions concerning non-equilibrium or “lag” phases in the distributional dynamics could be tested in vitro where the supply of susceptible host cells could be most easily manipulated.

### Host-Cell Heterogeneity—Intrinsic or Stochastic?

Our basic modelling approach has demonstrated that heterogeneities in intrinsic host-cell attributes are not a necessary condition for a heterogeneity in bacterial burden. However, we can use our model framework to ask whether the specific distributions of intracellular bacterial numbers observed in the tissues of infected mice provide evidence for any intrinsic heterogeneity in host cell permissiveness. Our best-fitting intrinsic heterogeneity models concluded that the burst thresholds are very variable across host cells. From these models we might conclude that there is considerable intrinsic heterogeneity among host cells in their response to infection. However, our parallel stochastic heterogeneity models provided even better fits to the bacterial distribution data, describing variation in burst events as purely stochastic consequences of uniform underlying processes of bacterial division, accumulation and consequent lysis.

A first conclusion of this parallel approach is that host cells do indeed vary in burst sizes and that both constitutive and stochastic interpretations of host heterogeneity are consistent with the observed biological data. Furthermore, exact equivalences can be found between some of the constitutive and stochastic approaches taken above, in terms of generating equivalent stationary distributions of bacteria (Protocol S1). However, there are important asymmetries in the equivalences between the two approaches: while equivalences can be found for some stochastic models in terms of constitutive “heterogeneous thresholds”, others (notably the best fitting density-dependent fission model) would create negative burst probabilities when interpreted as a “threshold” model. Therefore, the best fits to the data cannot be explained in terms of a heterogeneous threshold model. In contrast, meaningful equivalences can be found for all of the explored heterogeneous threshold models in terms of density-dependent fission models (Protocol S1). We therefore interpret our robust conclusion of heterogeneous bursts as a result of a density-independent stochastic burst process underlain by density-dependent division, and reject the intrinsic heterogeneity hypothesis.

### Strain Differences

We can now use the branching-process framework to focus on the distinct population dynamical signatures of the virulent (SL 5560) and attenuated (SL 3261) strains. Given a stochastic lysis function generating burst size *N*, changes in the underlying division rate *α* can account for the direction of change in population growth rate *γ* and in the distribution of bacteria between the attenuated and virulent strains. The attenuated strain SL3261 is an auxotrophic mutant, requiring for growth metabolites not abundant in vertebrate tissues. This handicap ensures that SL3261 propagates far more slowly than the virulent wildtype [7], and has led to its successful use as a live vaccine [22]. Our new results allow us to tease out the driving role of division rate *α* in shaping the ecological differences between these two strains.

A consequence of the dynamical distinctions between the virulent and attenuated strain is that the mean burst size is greater for the faster-dividing virulent strain. Specifically, using the density-dependent fission model, we can estimate the mean burst size (*R*_0_) for the virulent strain to be 7.16 whereas for the attenuated bacteria it is 3.25. Very simply, one implication of this difference is that to achieve a given density of bacteria within the host, the attenuated strain destroys more liver phagocytes – in other words the attenuated strain is relatively profligate in its use of host resources. In consequence, despite the attenuated strain growing at a slower rate, it destroys liver cells at a rate comparable to the virulent strain Therefore, if host pathogenicity was influenced, at least in part, by liver phagocyte death, then we would expect that an attenuated strain would be more damaging to the host than the virulent strain for equal bacterial numbers in the tissues. Of course, pathogenicity is also heavily influenced by the absolute bacterial numbers in the tissues that normally reach high levels in progressive infections with virulent bacteria. Generally, a consideration of the mean burst size of an intracellular pathogen coupled with the evaluation of absolute numbers in the tissues offers a novel perspective on the relationship between population growth rate and pathogenicity.

### Medical Intervention

The present debate over the usefulness of multiple-drug therapy is focused on the consequences for drug resistance [23,24]. Here we introduce a new focus on the importance of dynamical interactions between different classes of antimicrobials. Figure 4 illustrates that reducing extracellular survival *β* increases the profitability of reducing intracellular division rate *α*, and vice versa. This dynamical interaction has consequences for both the short- and long-term efficacy of multiple-drug therapy.

In the short-term, the dynamical interaction between *α*_0_ and *β* control offers a novel perspective on evaluating the efficacy of existing antibiotic treatments, and on the design of maximally-efficient combination therapies. In the longer-term, dynamical interactions between the two control points *α*_0_ and *β* can also make predictions on the evolutionary fate of specific resistance mutants. For example, given bacterial influence over host cell lysis parameters, we can consider how bacterial selection would act on lysis rates in response to a given intervention. Increasing extracellular control would select for reduced lysis rates – i.e. the evolved response to strong *β* intervention could be a form of refuge resistance, spending longer intracellularly to reduce exposure to control agent. In consequence, we would expect less dispersive infections with greater mean intensities per cell (and less susceptible to further extracellular control).

### Combining Dynamical and Mechanistic Approaches

Looking forward, an important challenge will be to integrate further the dynamical approach developed in this paper with the results of mechanistic molecular/physiological approaches to the biology of host pathogen interactions. Our current results suggest that in vivo, apoptosis is dynamically unimportant. Significant levels of apoptosis associated with bacterial death would be visible in our study in a reduction of the extracellular (or more generally, between-host cell) survival term *β* < 1, for which we found no evidence (unpublished data). Alternately, if bacteria were to remain viable within apoptotic bodies, and phagocytes ingesting apoptotic bodies were to become infected, then the bacteria have simply swapped one host cell for another, and the dynamics remain unchanged so long as the new cell lyses with the same constant probability. We therefore conclude that necrotic bursts, liberating infective bacteria, dominate the dynamics, relegating apoptosis to at best a marginal phenomenon. In support we note preliminary findings obtained via direct in vivo observation of the colocalization between apoptotic cells and bacteria indicate that apoptosis of infected cells is an extremely rare event in the livers of mice infected with wild type *Salmonella* (unpublished data).

Turning to host cell lysis, on the basis of observed distributions of bacteria in vivo, we make the striking prediction that the rate of infected cell burst is a constant (independent of density of bacteria within the host cell). S. enterica are known to induce cell death using secreted proteins encoded on the *Salmonella* pathogenicity islands 1 and 2 (SPI-1 and SPI-2) and also by production of cytotoxins [12]. Some of these molecules are known to be regulated by environmental factors [25], opening the possibility that the constant lysis rate reflects a density-dependent adjustment of individual microbe contribution to host-cell lysis, or alternately that the induction of lysis is a physiologically cheap and non-limiting step in microbial proliferation. We also predict a density-dependent reduction in fission rates within host cells, suggestive of a local (within cell) exhaustion of resources (nutrients and/or space), or even of a regulation of division via local accumulation of “quorum sensing” molecules [26]. The current models share a number of assumptions that could be relaxed in future theoretical studies, ideally developed in tandem with analyses of relevant in vivo data. For instance, introducing extracellular delays and a variable associated mortality could provide further insights into the dynamical impacts of extracellular versus intracellular and bacteriocidal versus bacteriostatic controls on infection.

In summary, we present a reductionist “demographic” null model upon which further complications (e.g. host cell heterogeneity) can be assessed. Our best-fitting model makes specific predictions on the intracellular dynamics of *Salmonella* fission and consequent cell lysis. For within-cell pathogens in general, we show that within-cell dynamics have implications across pathogen dynamics, evolution and control, and should be explored in future empirical and theoretical studies.

## Materials and Methods

For further details, see Protocol S1.

### Homogeneous threshold burst.

Here we define a single threshold burst function, where infected cells lyse only when the intracellular count reaches a fixed value *N* (Figure 1C). The dynamics of host cells *M_n_* infected by *n* bacteria is then given by

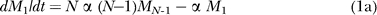
*dM*_1_/*dt* = *N α* (*N*−1)*M_N_*_-1_ − *α M*_1_(1a)


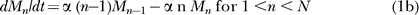
*dM_n_*/*dt* = α (*n*−1)*M_n_*_−1_ − α n *M_n_* for 1 <*n* < *N*(1b)



The expected distribution of infection intensities can be derived (see Protocol S1), converging for any reasonable initial condition (Σ*M_n_* finite) to *M_n_*∝*e^αt^Q_n_*, with

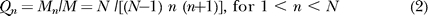
*Q_n_* = *M_n_*/*M* = *N* /[(*N*−1) *n* (*n*+1)], for 1 < *n* < *N*(2)
 where *M* is the total number of infected cells. Model 2 is plotted in Figure 2 (black line).

### Heterogeneous threshold burst.

Here the dynamics of host cells *M_n_* infected by *n* bacteria are described as for the homogeneous threshold model, except that the particular threshold *N* can vary among host cells with a prescribed distribution *D_N_* (eg Poisson). The resulting stationary distribution is (see Protocol S1)

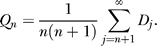



We study four specific cases: (i) Poisson *D_n_*=*e^−r^r^n^*/*n*! ; (ii) Gamma *D_n_*∝*n^a−1^e^−bn^ ;* (iii) Negative Binomial *D_n_*=*p^r^* (1−*p*)*^n^* Γ(*n+r*)/{ Γ (*n*+1) Γ (*r*)}; and (iv) Normal *D_n_*∝ exp (−*a* [*n − b*]^2^).

### Stochastic burst.

We now define a probabilistic burst function, with burst rate defined as *μ_n_* and bacterial fission rate defined as *α_n_* The dynamics of the number of host cells *M_n_* infected by *n* bacteria is given by

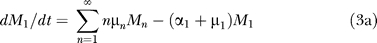


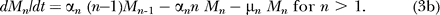
*dM_n_*/*dt* = α*_n_* (*n*−1)*M_n_*_-1_ − α*_n_n M_n_* − *μ_n_ M_n_* for *n* > 1. (3b)



The distribution of infection intensities rapidly converges to the quasistationary distribution *M_n_*∝*e^γt^Q_n_*, where the rate γ satisfies

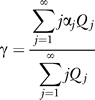
and the stationary distribution *Q_n_* = *M_n_*/*M* satisfies the recurrence relationship

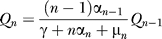
for *n* > 1 (see Protocol S1). When fission is density-independent, i.e. α = constant, we find *γ = α* and *Q_n_* can be computed directly. When *α_n_* depends on *n*, the equations for *γ* and *Q_n_* must be solved simultaneously. We assume that lysis is quadratic in *n*, *μ_n_* = *μ*_0_ + *μ*_1_ *n* + *μ*_1_ *n* ^2^, and fission decreases exponentially in *n*, *α = α*_0_ *e^−α_e_^^n^*.

### Medical intervention.

Given the best-fitting density-dependent fission model, the threshold value of the inter-cellular survival parameter *β* for which the disease is just controlled is given by (see Protocol S1)

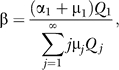
where the *Q_n_* are computed from the recurrence relation

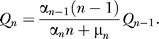



## Supporting Information

Protocol S1 Details of Model Constructions and Statistical Analyses (81 KB PDF)

## Abbreviations

AIC: Akaike information criterion
LSCFM: laser scanning confocal microscopy
MCFM: multicolour fluorescence microscopy
RNI: reactive nitrogen intermediate
ROI: reactive oxygen intermediate
SCV: Salmonella enterica-containing vacuole
SPI: Salmonella pathogenicity islands
