# Supplementary material for: Intracellular Demography and the Dynamics of Salmonella enterica Infections
Source: PLoS Biol. 2006 Oct 17;4(11):e349. doi: 10.1371/journal.pbio.0040349 (PMC1609125; doi:10.1371/journal.pbio.0040349)
Supplement: Supplementary file 1 [file pbio.0040349.sd001.pdf]

Protocol S1
Intracellular demography and the dynamics of *Salmonella enterica* infections (Brown, Cornell *et al.*)

1 Burst at a fixed threshold

Consider first a single phagocyte which is infected with a bacteria at time $t = 0$. By assumption, the cell will not be further infected by bacteria from outside the cell, and the number of bacteria increases by fission at per capita rate α until the threshold N is reached. Rather than solving directly Eqns. [1a,b] from the Methods section of the paper, an explicit solution may more straightforwardly be obtained from an alternative (but equivalent) formalism where individual cells are considered before scaling up to the whole population. The Forward Equation for the probability P_n that there are n bacteria in a cell at a time t after infection is

$$\frac{dP_n}{dt} = \alpha(n-1)P_{n-1} - \alpha n P_n, \qquad n \leq N-1$$

This can be solved by standard techniques such as generating functions, subject to the initial condition $P_1(t=0) = 1$, to yield

$$P_n(t) = e^{-\alpha t}(1 - e^{-\alpha t})^{n-1} \qquad n \leq N-1 \tag{1}$$

The cell lyses and releases N bacteria the instant that $n = N$, so the average rate of release of propagules at time t from a cell infected at time 0 is

$$R(t) = \alpha N(N-1)P_{N-1}(t). \tag{2}$$

Since all infections are the result of the release of propagules via cell burst, and these cells will themselves have been infected some time in the past, we can write a self-consistent equation for the rate $I(t)$ at which cells are being infected at time t:

$$I(t) = \int_{t_0}^{t} I(t')R(t-t')dt', \tag{3}$$

where t_0 is the time when the first cell was infected. This assumes that susceptible host cells are sufficiently in excess that each propagule successfully and instantaneously infects a new cell.

Let us assume that the initial infection was far enough in the past so that $t_0 \to -\infty$, and that the infection rate increases exponentially, $I = I_0 e^{\gamma t}$ (it is possible to justify this assumption by using Laplace transforms to solve Eqn. (3) explicitly, whereupon we need to to include an initial external infection source at t_0). Any reasonable initial state (i.e. one with a finite number of bacteria) will converge to this *quasistationary state* over time. Dividing eqn. (3) by $I(t)$ gives

$$1 \;=\; \int_{-\infty}^{t} e^{\gamma(t'-t)}R(t-t')dt'$$

$$
\begin{aligned}
&= \alpha N(N-1)\int_0^\infty e^{-\gamma s}e^{-\alpha s}(1-e^{-\alpha s})^{N-2}ds \\
&= N(N-1)B(\frac{\gamma}{\alpha}+1, N-1) \\
&= \frac{N(N-1)\Gamma(\frac{\gamma}{\alpha}+1)\Gamma(N-1)}{\Gamma(\frac{\gamma}{\alpha}+N)} \qquad (4)
\end{aligned}
$$

where we have substituted for R from Eqn. (2) and P_{N-1} from Eqn. (1), and B and Γ are respectively the Beta and Gamma functions (Abramowitz M, Stegun I, *Handbook of Mathematical Functions* [Dover, New York, 1972]; $\Gamma(a) \equiv \int_0^\infty y^{a-1}e^{-y}dy$; $B(a,b) \equiv \int_0^1 y^{a-1}(1-y)^{b-1}dy = \frac{\Gamma(a)\Gamma(b)}{\Gamma(a+b)}$). The Gamma function has the property $\Gamma(y+1) = y\Gamma(y)$, and for integer N reduces to $\Gamma(N) = (N-1)!$. We can therefore further manipulate Eqn. (4):

$$
\begin{aligned}
1 &= \frac{N(N-1)\Gamma(\frac{\gamma}{\alpha}+1)\Gamma(N-1)}{\Gamma(\frac{\gamma}{\alpha}+N)} \\
&= \frac{\Gamma(\frac{\gamma}{\alpha}+1)N!}{\Gamma(\frac{\gamma}{\alpha}+N)} \\
&= \frac{\Gamma(\frac{\gamma}{\alpha}+1)N!}{(\frac{\gamma}{\alpha}+N-1)(\frac{\gamma}{\alpha}+N-2)\cdots(\frac{\gamma}{\alpha}+1)\Gamma(\frac{\gamma}{\alpha}+1)} \\
&= \frac{N!}{(\frac{\gamma}{\alpha}+N-1)(\frac{\gamma}{\alpha}+N-2)\cdots(\frac{\gamma}{\alpha}+1)}.
\end{aligned}
$$

Since the denominator is a monotonically increasing function of $\frac{\gamma}{\alpha}$, there can be only one solution to this equation, which is clearly $\gamma = \alpha$. This makes sense intuitively, because in a quasistationary state we would expect the total number of infected cells to be proportional to the total bacterial population size, which is itself proportional to $e^{\alpha t}$ since there is no source of bacterial mortality.

The total number of infected cells with n bacteria will be $\int_{-\infty}^t I(t')P_n(t-t')dt'$, while the total number of infected cells will be proportional to $I(t)$. The fraction of cells which have n bacteria will be

$$
\begin{aligned}
Q_n &\propto \frac{\int_{-\infty}^t I(t')P_n(t-t')dt'}{I(t)} \\
&= \int_{-\infty}^t e^{\alpha(t'-t)}e^{-\alpha(t-t')}(1-e^{-\alpha(t-t')})^{n-1}dt' \\
&\propto B(1,n) \\
&= \frac{\Gamma(2)\Gamma(n)}{\Gamma(n+2)} \\
&= \frac{1}{n(n+1)}.
\end{aligned}
$$

We can straightforwardly prove by induction that $\sum_{n=1}^{N-1}\frac{1}{n(n+1)} = 1-\frac{1}{N}$, so the correctly normalised form for $Q_n$is

$$
Q_n = \frac{N}{(N-1)n(n+1)}
$$

2 Heterogeneous burst size

Suppose now that the pre-programmed threshold N at which each cell lyses differs between cells. Let D_N be the burst size distribution, i.e. the fraction of cells that have

threshold N (so $\sum_N D_N = 1$). The probability that a cell contains n bacteria a time t after it was infected still satisfies Eqn. (1), but the mean rate of release of propagules is now

$$R(t) = \alpha \sum_N D_N N(N-1) P_{N-1}(t).$$

The infection rate $I(t)$ satisfies Eqn. (3), and again looking at the quasistationary state where $t_0 \to -\infty$ and $I(t) = I_0 e^{\gamma t}$ we find

$$\begin{aligned} 1 &= \int_{-\infty}^{t} e^{\gamma(t'-t)} R(t-t') dt' \\ &= \sum_N D_N N(N-1) B(\frac{\gamma}{\alpha}+1, N-1). \end{aligned}$$

As before, we find that this equation has only a single solution, $\alpha = \gamma$, because $B(\frac{\gamma}{\alpha} + 1, N-1)$ is a monotonically decreasing function of $\frac{\gamma}{\alpha}$.

The steady state distribution of bacteria between cells satisfies

$$Q_n \propto \frac{\int_{-\infty}^{t} I(t') \sum_{N=n+1}^{\infty} D_N P_n(t-t') dt'}{I(t)},$$

where the limits on the sum over N arise because $P_n = 0$ for $n > N$ (see Eqn. (1)). Substituting for P_n from Eqn. (1) and integrating we get

$$Q_n \propto \frac{1}{n(n+1)} \sum_{N=n+1}^{\infty} D_N. \tag{5}$$

Eqn. (5) allows us to compute the steady state distribution for a given distribution D_N. The cases we studied were (i) Poisson $D_N = e^{-r}\frac{r^N}{N!}$ (ii) Bimodal $D_N = r\delta_{N,N_1} + (1-r)\delta_{N,N_2}$(iii) Gamma $D_N \propto N^{a-1} e^{-bN}$ (iv) Negative Binomial $D_N = \frac{\Gamma(N+r)}{\Gamma(N+1)\Gamma(r)} p^r (1-p)^N$ (v) Normal $D_N \propto \exp[-a(N-b)^2]$.

3 Density-dependent lysis and fission

Let us now assume that cells lysis takes place at a given rate, rather than instantaneously at a threshold. Let this rate μ_n depend on the number n of bacteria in the cell. Let us also assume that bacterial fission is density dependent, with α_n being the per-capita splitting rate for a bacteria in a cell containing n bacteria. While the approach of sections 1 and 2 can be used to give an explicit closed-form solution for some simple cases, we instead develop a method for computing the distribution numerically for general α_n and μ_n. The Forward equation for the total number M_n of cells with n bacteria at time t is

$$\frac{dM_n}{dt} = \alpha_{n-1}(n-1)M_{n-1} - \alpha_n n M_n - \mu_n M_n + \delta_{n1} \sum_{j=1}^{\infty} j \mu_j M_j, \tag{6}$$

where $\delta_{n,1}$ is the Kroeneker delta which ensures that the final term (which represents the infection of susceptible cells) only applies to $n = 1$.

If we assume a quasi-stationary state with $M_n \propto e^{\gamma t} Q_n$, Eqn. (6) becomes

$$\gamma Q_n = \alpha_{n-1}(n-1)Q_{n-1} - \alpha_n n Q_n - \mu_n Q_n + \delta_{n1} \sum_{j=1}^{\infty} j \mu_j Q_j. \tag{7}$$

For $n \geq 2$, this can be rearranged to give a recurrence relation for the distribution of bacteria between cells:

$$Q_n = \frac{\alpha_{n-1}(n-1)}{\gamma + \alpha_n n + \mu_n} Q_{n-1}. \tag{8}$$

Meanwhile, multiplying Eqn. (7) by n and summing gives an expression for γ:

$$\begin{aligned} \gamma \sum_j j Q_j &= \sum_{j=1}^{\infty} \alpha_{j-1} j(j-1) Q_{j-1} - \sum_{j=1}^{\infty} \alpha_j j^2 Q_j - \sum_{j=1}^{\infty} j\mu_j Q_j + \sum_{j=1}^{\infty} j\mu_j Q_j \\ &= \sum_{j=1}^{\infty} j\alpha_j Q_j \\ \Rightarrow \gamma &= \frac{\sum_{j=1}^{\infty} j\alpha_j Q_j}{\sum_j j Q_j}. \end{aligned} \tag{9}$$

For the case where fission is density-independent, $\alpha_n = \alpha$, Eqn. (9) reduces to $\gamma = \alpha$, so it is possible to compute the Q_n directly (initially in terms of Q_1, then the whole distribution may be normalised to sum to unity) from Eqn. (8) in the form

$$Q_n = \frac{(n-1)}{1 + n + \frac{\mu_n}{\alpha}} Q_{n-1}. \tag{10}$$

Note that the stationary distribution Q_n is only sensitive to the ratio $\frac{\mu_n}{\alpha}$, not to the absolute values of these rates.

If fission is density dependent, Eqns. (8) and (9) need to be solved self-consistently. One convergent procedure is to assume a value of γ, compute Q_n from Eqn. (8), calculate an updated γ using Eqn. (9), and iterate until the initial and final values of γ are numerically indistinguishable. The distribution Q_n is only sensitive to the ratios $\frac{\alpha_n}{\gamma}$ and $\frac{\mu_n}{\alpha_n}$, so our quoted values of μ_n and γ are in units of α_0.

3.1 Control of within-host proliferation

If there is inter-cellular mortality of bacteria, so that only a fraction β successfully reinfect, then Eqn. (6) is replaced by

$$\frac{dM_n}{dt} = \alpha_{n-1}(n-1)M_{n-1} - \alpha_n n M_n - \mu_n M_n + \beta\delta_{n1} \sum_{j=1}^{\infty} j\mu_j M_j. \tag{11}$$

The threshold for control of within-host proliferation is obtained by setting $\frac{dM_n}{dt} = 0$. The stationary distribution for a given function α_n is then

$$Q_n = \frac{\alpha_{n-1}(n-1)}{\alpha_n n + \mu_n} Q_{n-1}$$

for $n \geq 2$. The threshold value of β is then obtained by setting $n = 1$ in Eqn. (11)

$$\beta = \frac{(\alpha_1 + \mu_1)Q_1}{\sum_{j=1}^{\infty} j\mu_j Q_j}$$

4 Equivalence between models

Since we have data for the stationary distribution of bacteria between cells, we can only distinguish between models via their predictions for Q_n. We have had to assume

specific parametric forms for either the burst size distribution D_N or the lysis and fission functions μ_n and α_n. Does the difference in quality of fit between models reflect true differences between the classes of models, or is it merely an artefact of our chosen parametrisations?

First, let us show that, for any heterogeneous burst model characterised by D_N, there is a density-dependent lysis model characterised by μ_n that gives exactly the same prediction for Q_n. First, using Eqn. (5) we find the following expression for a heterogeneous burst model (for $n \geq 2$):

$$\frac{\sum_{j=n+1}^{\infty} D_j}{\sum_{j=n}^{\infty} D_j} = \frac{(n+1)Q_n}{(n-1)Q_{n-1}}.$$

For a density-dependent lysis model with density-independent fission, Eqn. (10) can be written in the form

$$\frac{Q_n}{Q_{n-1}} = \frac{(n-1)}{1+n+\frac{\mu_n}{\alpha}}.$$

Combining these two expressions, we find

$$\begin{aligned}
\frac{(n+1)}{1+n+\frac{\mu_n}{\alpha}} &= \frac{\sum_{j=n+1}^{\infty} D_j}{\sum_{j=n}^{\infty} D_j} \\
\Rightarrow 1+n+\frac{\mu_n}{\alpha} &= \frac{(n+1)\sum_{j=n}^{\infty} D_j}{\sum_{j=n+1}^{\infty} D_j} \\
\Rightarrow \frac{\mu_n}{\alpha} &= \frac{(n+1)\sum_{j=n}^{\infty} D_j}{\sum_{j=n+1}^{\infty} D_j} - (n+1) \\
&= (n+1)\frac{\sum_{j=n}^{\infty} D_j - \sum_{j=n+1}^{\infty} D_j}{\sum_{j=n+1}^{\infty} D_j} \\
&= \frac{(n+1)D_n}{\sum_{j=n+1}^{\infty} D_j}. \qquad (12)
\end{aligned}$$

Eqn. (12) allows us to compute the lysis function μ_n for a density-dependent lysis model that is equivalent to a given heterogeneous burst model defined by D_N. Since all the D_n are positive, the μ_n are also positive, so the lysis model is biologically meaningful. Therefore, for any heterogeneous burst model with a given number of parameters, we can construct an equivalent density-dependent lysis model with the same number of parameters that gives exactly the same fit. Figure 1 shows the computed lysis functions μ_n equivalent to the maximum likelihood fit for the heterogeneous burst model with Gamma-distributed burst size distribution. It is also possible to show that there is a density-dependent fission model that is equivalent to each heterogeneous burst model, though the proof is a little harder.

However, the fact that we can find a model of class 'A' (say) which is equivalent to any given model of class 'B' (say) does not logically mean that there is a model of class B which is equivalent to any model of class A — in other words, the set of models of class B might merely correspond to a subset of the models of class A. Let us now see if there is an equivalent heterogeneous burst model to any given density-dependent fission model, the class which gave us the best numerical fit. Considering Eqn. (5) for $j = n$ and $j = n-1$

$$\begin{aligned}
D_j &= j(j-1)Q_{j-1} - j(j+1)Q_j \\
&= j(j-1)Q_{j-1}\left(1 - \frac{(j+1)Q_j}{(j-1)Q_{j-1}}\right) \\
&= j(j-1)Q_{j-1}\frac{(\gamma+\mu+n\alpha_n-(n+1)\alpha_{n-1})}{\gamma+\mu+n\alpha_n}, \qquad (13)
\end{aligned}$$

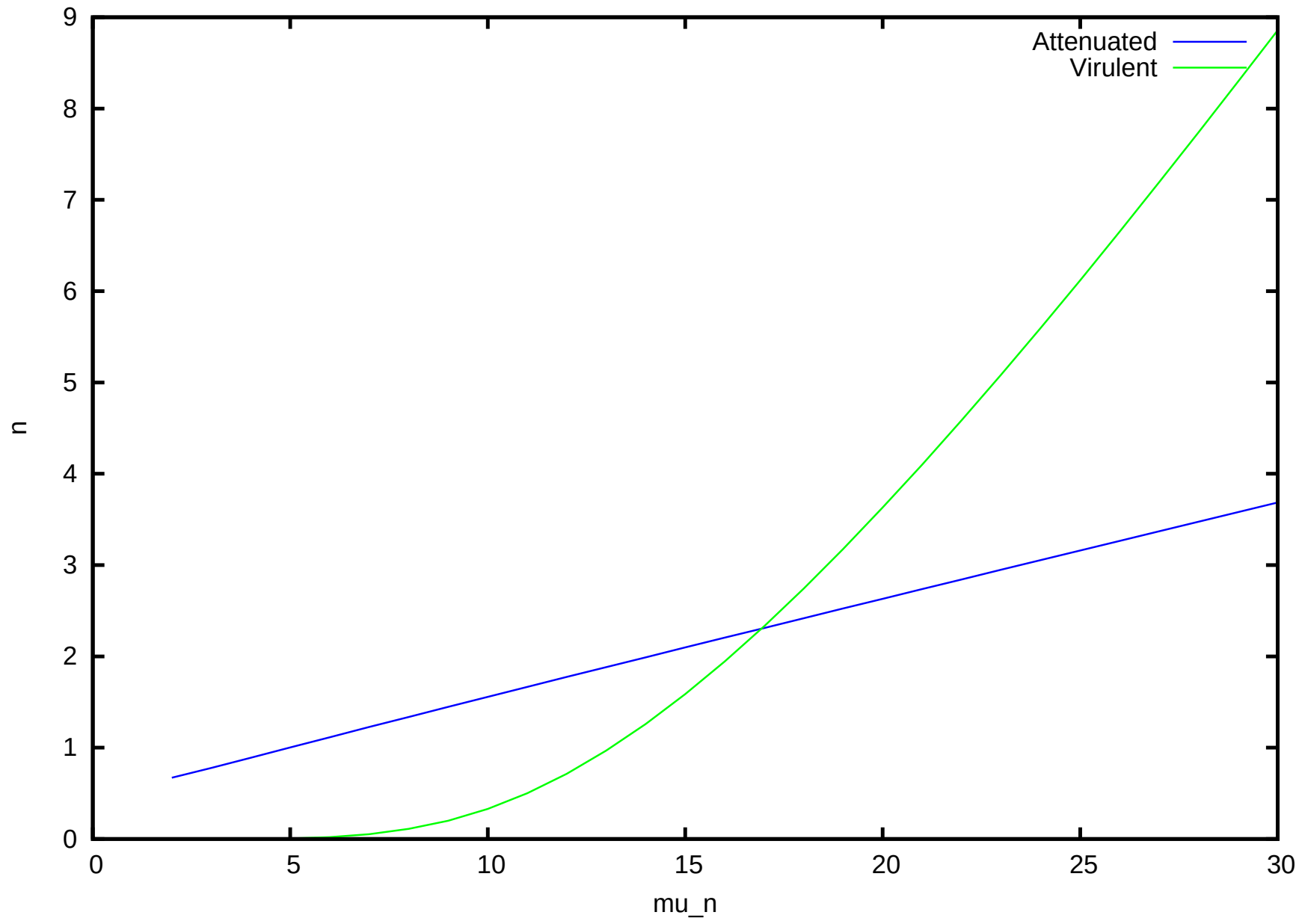

Figure 1: Lysis functions μ_n computed from Eqn. (12) for lysis models equivalent to the best fit heterogeneous burst model with Gamma-distributed burst size distribution.

where in the last line we have substituted $\frac{Q_j}{Q_{j-1}}$ for a density-dependent fission model from equation (8), assuming density-independent lysis $\mu_n = \mu$. While this formula allows us to compute D_n from α_n, it turns out that for some fission functions α_n the burst distribution D_n is sometimes negative—which means that the 'equivalent' heterogeneous burst model is not biologically valid. This is the case for the best-fit density-dependent fission model for the virulent strain with exponentially decreasing fission, as illustrated in Figure 2. Conversely, using the same method we can show that, for any given density-dependent lysis model with density-*independent* fission, there is indeed a valid equivalent heterogeneous burst model (we already proved the equivalence in the opposite direction in Eqn. (12)).

It is intuitively reasonable (though mathematically non-trivial) that heterogeneous burst models and density-dependent lysis models give rise to the same class of stationary distributions. Until the cell bursts, the dynamics are the same for both models — the difference being that the size at which burst takes place is pre-determined in the former case and emerges dynamically from the lysis function in the latter. However, for the density-dependent fission model the rate of bacterial splitting itself slows down over time, so there is no reason to expect the dynamics to be equivalent to the other models.

We conclude that, while there is a degree of equivalence between models, the models with density-dependent fission occupy a larger 'space' than the others in the sense that there is a density-dependent fission model equivalent to each heterogeneous burst model but not vice versa. It so happens that no heterogeneous burst model is able to give the excellent fit to the virulent strain found for our best density-dependent fission model, allowing us to reject the former class of model in favour of the latter.

5 Statistical procedure for model fitting

The output of the models is a stationary distribution $Q_n(X)$ of the number of bacteria per cell, which depends on a set of parameters X and is to be compared with observed

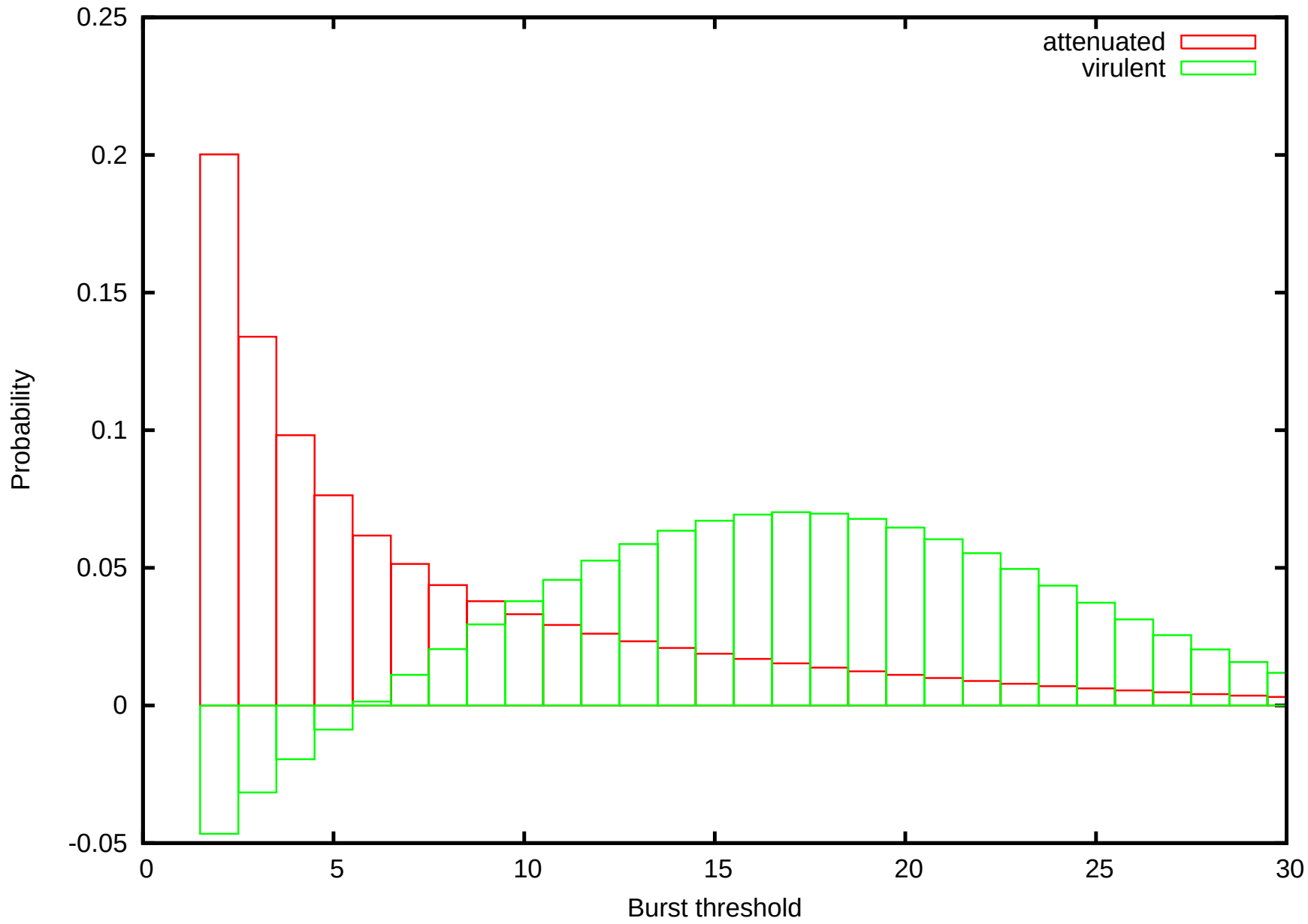

Figure 2: Burst size distribution for the heterogeneous burst model equivalent to the best fit density-independent lysis model with exponentially decreasing fission, computed from Eqn. (13). Note Q_n is negative for the virulent strain when $n < 6$.

counts C_n of the number of cells with n bacteria. If $N = \sum C_n$ is the total number of counts, then assuming independence of observations the $C_n$will be multinomially distributed with likelihood

$$L(\{C_n\}|\{Q_n\}) = N! \prod_n \frac{Q_n^{C_n}}{C_n!}.$$

We used a combination of conjugate gradient and simplex methods to iterate towards values of the parameters X which gave distributions Q_n that maximised the likelihood L for our data C_n. The numerical routines were written in Fortran 90, based on the algorithms in Press *et al.* (*Numerical Recipes in FORTRAN: the art of computer programming*, Cambridge University Press, available online at `http://www.nr.com`). The Akaike Information Criterion is calculated as $AIC = -2 \ln L + 2k$, where k is the number of fitted parameters in the model.
